# Pandemic Preparedness: Analyzing National Plans for Respiratory Pathogen Pandemics in the Americas Region

**DOI:** 10.1093/infdis/jiaf047

**Published:** 2025-03-10

**Authors:** Andrea Patricia Villalobos Rodríguez, Iyanna Wellington Perkins, Shoa Moosavi, Ana de la Garza, Ángel Rodríguez, Britney McMurren, Juliana Almeida Leite, Jairo Méndez-Rico, Lidia Redondo-Bravo, Miriam Esther Blanco Reyes, Olaya Astudillo, Paula Couto, Priscila S Born, Sarah Hess, Shoshanna Goldin, Hannah Lewis, Tamara Mancero, Andrea S Vicari, Marc Rondy

**Affiliations:** Health Emergencies Department, Pan American Health Organization, Washington, District of Columbia, USA; Health Emergencies Department, Pan American Health Organization, Washington, District of Columbia, USA; Health Emergencies Department, Pan American Health Organization, Washington, District of Columbia, USA; Health Emergencies Department, Pan American Health Organization, Washington, District of Columbia, USA; Health Emergencies Department, Pan American Health Organization, Washington, District of Columbia, USA; Epidemic and Pandemic Preparedness and Prevention, World Health Organization, Geneva, Switzerland; Health Emergencies Department, Pan American Health Organization, Washington, District of Columbia, USA; Health Emergencies Department, Pan American Health Organization, Washington, District of Columbia, USA; Health Emergencies Department, Pan American Health Organization, Washington, District of Columbia, USA; Health Emergencies Department, Pan American Health Organization, Washington, District of Columbia, USA; Health Emergencies Department, Pan American Health Organization, Washington, District of Columbia, USA; Health Emergencies Department, Pan American Health Organization, Washington, District of Columbia, USA; Health Emergencies Department, Pan American Health Organization, Washington, District of Columbia, USA; Epidemic and Pandemic Preparedness and Prevention, World Health Organization, Geneva, Switzerland; Epidemic and Pandemic Preparedness and Prevention, World Health Organization, Geneva, Switzerland; Epidemic and Pandemic Preparedness and Prevention, World Health Organization, Geneva, Switzerland; Health Emergencies Department, Pan American Health Organization, Washington, District of Columbia, USA; Health Emergencies Department, Pan American Health Organization, Washington, District of Columbia, USA; Health Emergencies Department, Pan American Health Organization, Washington, District of Columbia, USA

**Keywords:** pandemic preparedness, respiratory diseases, pandemic preparedness and response plans, IHR, monitoring and evaluation

## Abstract

**Background:**

To identify gaps and define priority actions to strengthen national pandemic preparedness and response plans, we assessed the concordance between national plans for respiratory pathogens against the World Health Organization (WHO) pandemic checklist and the States Parties Annual Report (SPAR) in the Americas.

**Methods:**

In this retrospective, semiquantitative study, we reviewed the most recent respiratory pandemic plans for 35 Pan American Health Organization (PAHO) member states and assessed their concordance with (1) actionable guidelines in WHO pandemic checklist and (2) International Health Regulations (IHR) (2005) core capacities using the latest SPAR tool. We developed 25 tracking questions to identify gaps, strengths, and opportunities for improvement using a 5-point scale. We compared the average SPAR score and the Pandemic Plans score to assess areas of convergence and variance between preparedness and capacities.

**Results:**

We analyzed 35 respiratory pandemic plans (2005–2024): 29 were influenza specific, 5 were COVID-19 specific, and 1 was not pathogen specific. Most national plans showed limited alignment with the content recommended in the pandemic checklist. The lowest concordance between plans and checklist was in public health and social measures (80% of plans with score of 1); emergency, logistics and supply chain management (74%); and research and development (71%). Conversely, the strongest subcomponents were policy, legal, and normative instruments (45% of plans with score 4 or 5); coordination (46%); and surveillance: early detection and assessment (43%).

**Conclusions:**

It is recommended that countries build on the strengths of their national pandemic preparedness and response plans and update them using PRET module 1. This will support countries advance the capacities required by the IHR.

National planning and preparation for pandemics were thoroughly tested by the coronavirus disease 2019 (COVID-19) pandemic, revealing both strengths and weaknesses in countries' responses [1]. The lasting impact of the COVID-19 pandemic on public health services highlights the importance of ensuring resilience and readiness for future pandemics [2]. In this context, resilience can be defined as the ability of a country to adapt to evolving conditions during an emergency and leverage response efforts for sustained health system improvements as the pandemic subsides [1]. The International Health Regulations (IHR 2005) is a legally binding document that requires its 196 States Parties to develop their capacities to rapidly detect, assess, notify, and respond to public health events of international concern [3]. The IHR States Parties Self-Assessment Annual Reporting tool (SPAR) [4] is part of the IHR (2005) monitoring and evaluation framework intended to support States Parties in fulfilling these obligations [5]. The submission of IHR annual reports using the SPAR tool allows the World Health Organization’s (WHO's) secretariat to compile a consistent report on the global state of health emergency preparedness for the World Health Assembly.

The latest SPAR tool (second edition) was published in 2021, and it contains 15 capacities and 35 indicators [5]. These capacities and indicators are directly related to a country's preparedness for all hazards, including respiratory pathogen pandemics, and the data provide valuable insights into real-world capacities and readiness for managing public health emergencies. Countries should have preparedness plans that document the necessary system structures (eg, health care infrastructure, supply chains, etc.), emergency preparedness actions (eg, strengthening surveillance systems and early warning mechanisms, training health care personnel, etc.), and roles and responsibilities of multisectoral actors to operationalize these core capacities in a health emergency. However, despite the availability of detailed preparedness plans, there are often discrepancies between these plans and the capacities recommended within the SPAR framework [5–7]. These discrepancies highlight the gaps between theoretical preparedness and operational readiness, as well as specific capacities required to respond to pandemics, which may not be fully captured by SPAR/IHR assessments (eg, safe management of a dead body, health emergency response: research, development and innovation). The WHO Preparedness and Resilience for Emerging Threats (PRET) initiative has been created to address these gaps and strengthen preparedness and functional capacities [8].

The PRET initiative aims to enhance global preparedness and resilience against emerging health threats. It focuses on using a mode-of-transmission approach to prepare and plan for pandemics [9]. PRET Module 1 focuses on the respiratory mode of transmission [8]. Using PRET Module 1 and its associated checklist for respiratory pathogen pandemic preparedness planning (for the purpose of this article, the pandemic checklist refers to the WHO checklist for pandemic preparedness planning [10]), countries can update their national pandemic plans to include actions that are more broadly applicable to respiratory pathogens, as well as pathogen-specific actions for key priority pathogens (eg, influenza viruses and coronaviruses) where needed/applicable. This is an alternative to a country having multiple pandemic plans, each only addressing a single pathogen. Additionally, pathogen-specific plans do not account for novel pathogens. Importantly, the pandemic checklist provides recommended content and key actions included in the IHR portal [6] and is therefore aligned with strengthening IHR core capacities.

Since the 2009 Influenza A H1N1 pandemic, there have been examples of assessments of national pandemic plans against benchmarks, such as a study published in 2012 of Ghana's plan (stated by the authors to be reflective of the sub-Saharan African setting) and another published in 2019 on European plans [11, 12]. Both studies found inconsistencies between the national plans and the actual preparedness capacities of the countries. More recently, a study published in 2024 assessed European national pandemic preparedness plans using the Health System Performance Assessment Framework for Universal Health Coverage. The authors found gaps in those plans, such as the lack of implementation strategies aimed at strengthening health financing or health workforce [13]. While studies have been done assessing pandemic plans in the Americas prior to the 2009 influenza pandemic, to our knowledge, there have been no published studies assessing respiratory pandemic preparedness and response plans in the Americas region in the past 15 years [14, 15].

The objective of this study was to assess national pandemic preparedness and response plans of countries in the Americas against key components of the pandemic checklist (which builds on the capacities and capabilities described in the WHO's PRET Module 1) and SPAR indicators, with the aim to identify gaps and define priority actions for strengthening these plans.

## METHODS

### Study Design

This retrospective, semiquantitative study, conducted in August 2024, aimed to use an internally developed questionnaire to assess concordance between countries' respiratory pandemic preparedness and response plans and 2 complementary approaches to pandemic planning: the first approach is the recommended actions outlined in WHO's PRET Module 1 and the accompanying pandemic checklist and, the second, the IHR core capacities using the latest SPAR tool (2021).

### Data Sources

#### Selection and Screening of National Respiratory Pandemic Preparedness and Response Plans

We compiled a dataset of respiratory pandemic preparedness and response plans from 35 member states of the Pan American Health Organization (PAHO) from 2005 to 2024. These plans were collected through PAHO's landscape surveys carried out in 2014, 2017, 2019, 2021, and 2023. We excluded plans if they lacked preparedness information (eg, if they focused exclusively on response) or if they focused solely on a single component of PRET Module 1, such as clinical management. We reviewed the remaining plans and selected the most recent plan from each country.

#### IHR Core Capacities 2023

For this study, we used data from the 2023 electronic State Parties Self-Assessment Annual Reporting Tool (e-SPAR), submitted by 35 countries in the Americas region, updated as of 21 May 2024. The e-SPAR tool is based on the country's assessment of the level of performance on each of the indicators using a scale of 1–5. Countries submit their self-assessed score for each indicator using a 5-point scale, which the e-SPAR converts to a percentage. For example, a score of 3 out of 5 would be converted to 60%. For this study, we used the score generated by the e-SPAR directly from country submissions for the analysis.

### Design of the Analytical Tool

#### Mapping Pandemic Checklist to SPAR/IHR Indicators

The first step in designing the analytical tool for this study was mapping the pandemic checklist components and subcomponents to the SPAR/IHR core capacities and indicators to ensure consistency and allow for comprehensive assessment of each plan's contents across both the PRET and the IHR approaches. This mapping enabled a structured analysis of preparedness elements, facilitating the identification of gaps and strengths. The pandemic checklist has 6 components and 25 subcomponents labeled A to Y (Table 1). The SPAR consists of 15 capacities and 35 indicators. In this analysis, 3 capacities (capacity 13 food safety, capacity 14 chemical events, and capacity 15 radiation emergencies) and their corresponding indicators were excluded as they were not relevant to the focus of this study.

**Table 1.** Mapping of Pandemic Checklist to SPAR Capacities and Indicators

Pandemic Checklist		IHR Core Capacities and SPAR Indicators	
Component	Subcomponent	IHR Core Capacities (2nd Edition)	SPAR Indicators (2nd Edition)
1. Emergency coordination	A. Planning	C7 Health emergency management	C7.1
	B. Policy, legal, and normative instruments	C1 Policy, legal, and normative instruments to implement IHR	C1.1
	C. Coordination	C2 IHR coordination and national IHR focal point functions and advocacy	C2.1, C2.2
	D. Financing	C3 Financing	C3.1, C3.2
	E. Human resources	C6 Human resources	C6.1, C6.2
	F. Guiding principles, gender and ethical considerations	C1 Policy, legal, and normative instruments to implement IHR	C1.2
2. Collaborative surveillance	G. Surveillance: overarching system considerations	C5 Surveillance	C5.2
	H. Surveillance: early detection and assessment	C5 Surveillance	C5.1
	I. Surveillance: monitoring circulating pathogen and use human health interventions	C5 Surveillance	C5.2
	J. Laboratory	C4 Laboratory, C7^a^ Health emergency management	C4.1, C4.2, C4.3, C4.4, C4.5, C7.3
	K. One Health/zoonotic disease: collaborative efforts	C2^a^ IHR coordination and national IHR focal point functions and advocacy, C12 Zoonotic diseases	C2.1, C12.1
3. Community protection	L. Public health and social measures: overarching	C10^a^ Risk communication and community engagement	C10.1
	M. Public health and social measures: community	C10^a^ Risk communication and community engagement	C10.3
	N. Border health and points of entry	C11 Points of entry and border health	C11.1, C11.2, C11.3
	O. Risk communication and community engagement	C10 Risk communication and community engagement	C10.2
4. Clinical care	P. Health service provision: continuity of essential health services	C8 Health services provision	C8.1, C8.2, C8.3
	Q. Health service provision: case management	C8 Health services provision	C8.1
	R. Infection prevention and control	C9 Infection prevention and control	C9.1, C9.2, C9.3
	S. Safe management of a dead body	^b^	^b^
5. Access to countermeasures	T. Health emergency response: emergency logistics and supply chain management	C7 Health emergency management	C7.3
	V. Equitable access, needs-based allocation, and medical countermeasures deployment for pandemic products such as vaccines and antivirals—NDVP planning	C8^a^ Health services provision	C8.3
	W. Health emergency response: essential medicines, products and materials	C7^a^ Health emergency management	C7.3
	X. Health emergency response: research, development, and innovation	^b^	^b^
6. Monitoring and evaluating, testing and revising plans	Y. Monitoring and evaluation	C7 Health emergency management	C7.1
	Z. Testing and revising plans	C7 Health emergency management	C7.2

Abbreviations: IHR, International Health Regulations (2005); NDVP, national deployment and vaccination plan; SPAR, States Parties self-assessment annual reporting tool.

^a^Additional capacity alignment included by the authors.

^b^No capacity or indicator related to this component and the subcomponents of the pandemic checklist was found.

We linked each subcomponent of the pandemic checklist to the relevant IHR core capacities by assigning 1 or more SPAR indicators to each subcomponent (Table 1). All SPAR indicators were assigned, except C2.3 (advocacy for IHR implementation), which did not have a direct equivalent in the pandemic checklist. In total, 12 capacities and 31 indicators were mapped. The alignment between the subcomponents of the pandemic checklist and the first 12 core capacities of the SPAR (2021) was carefully maintained, in accordance with the descriptions provided in Annex 1 of the pandemic checklist. Indeed, Annex 1 of the checklist contains all-hazard actions taken from the IHR benchmarks, which are in turn mapped to the SPAR capacities.

#### Formulation of the Tracking Questions to Analyze Respiratory Pandemic Preparedness and Response Plans

We formulated 25 tracking questions to identify gaps, strengths, and opportunities for improvement in the respiratory pandemic preparedness and response plan. Each question corresponded to a subcomponent of the pandemic checklist (Supplementary Table 1). These questions were designed to evaluate each plan's content and provide a score based on the semiquantitative 5-point scale we developed.

The 5-point scale was intended to measure the depth of description of each subcomponent in the plan. The rating score for pandemic plans (PP score) was as follows: 1 = the subcomponent was neither mentioned nor described (0%); 2 = the subcomponent was mentioned briefly in some part of the document but not described further (25%); 3 = the subcomponent was described with limitations (some of the criteria of the subcomponent are missing) (50%); 4 = the subcomponent was fully described for the national level only (75%); 5 = the subcomponent was described for all relevant levels (national, provincial, and local) and/or updated based on the latest recommendations (100%). Low scores indicated opportunities for more detailed and/or actionable descriptions of the relevant component.

### Data Extraction

Each national plan was initially reviewed, assessed, and scored by a single dedicated primary reviewer (MEBR) who had not previously been exposed to these plans, to minimize potential bias, and who was proficient in the original language of the plans (Spanish, English, French, and Portuguese). Three expert secondary reviewers (APVR, IW, SM) validated the primary reviewer's scores for a systematically selected subset of 7 plans with at least 1 plan per subregion (20%). All reviewers possess proficiency in the original language of the plans.

### Data Analysis

Results from the assessment and scoring process were tabulated in Microsoft Excel. An average PP score per subcomponent and component was calculated for each country. Additionally, countries were assigned a SPAR score for each subcomponent (in percent), by averaging the scores for SPAR indicators relevant to both the subcomponent and the component they aligned with.

For each component, we compared the average SPAR score reported by the country in terms of preparedness for emergencies and the PP score based on the reviewer's assessment of the preparedness capacities in relation to respiratory viruses. Through this comparison, we aimed to describe areas of convergence and variance between preparedness and capacities, as well as to identify strengths and weaknesses of each.

All statistical analyses and data visualizations were done using R (version 4.4.1; The R Foundation for Statistical Computing, 2024), using the R Studio integrated development environment (version 2024.04.2; Posit Software, 2024).

## RESULTS

### Reviewed Plans and Access to Information

**Figure 1.**
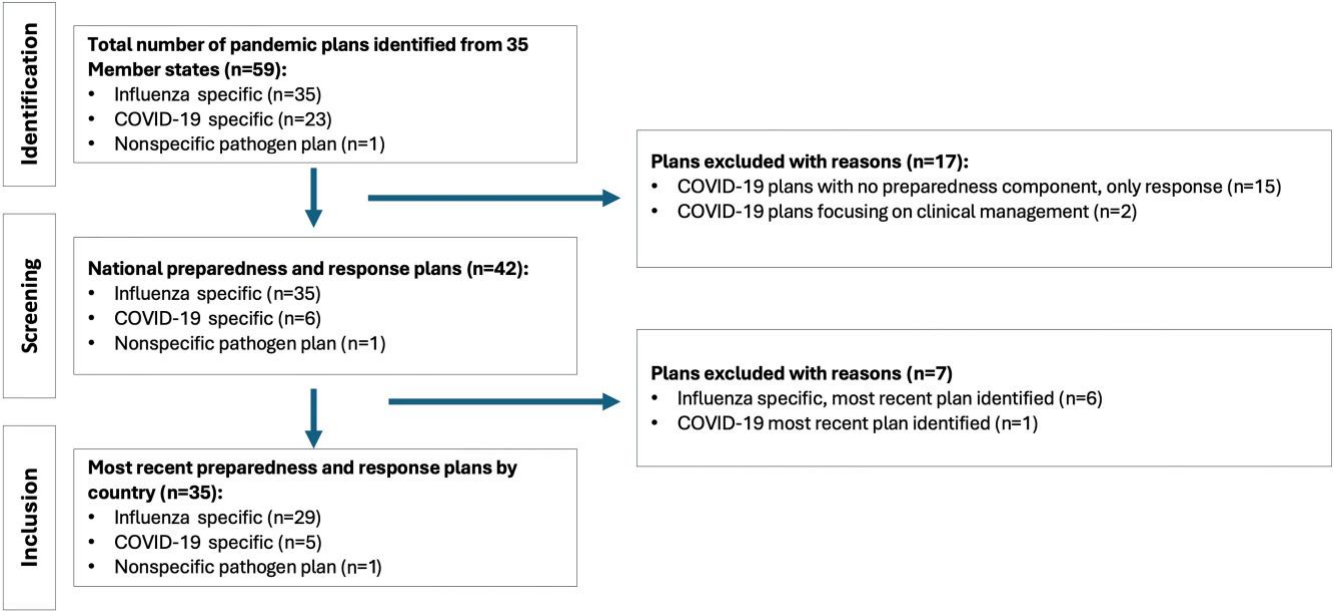
Steps in the respiratory pandemic plan selection process.

We compiled a dataset of 59 respiratory pandemic preparedness and response plans from all 35 member states of PAHO from 2005 to 2024. Of the 59 plans, 36 (61%) were accessible online. Among them, 35 were specific to influenza, 23 were specific to COVID-19, and 1 was a nonspecific pathogen plan (Figure 1). We excluded 17 plans because they either lacked preparedness information or focused solely on a single component of PRET Module 1. We reviewed the remaining 42 plans and selected the most recent plan from each country. In total, 35 country plans were included in the analysis. Supplementary Table 2 provides details about these plans. Regarding their scope, 29 were specific to influenza, 5 were specific to COVID-19, and 1 was nonspecific pathogen plans.

### Identification of Weaknesses and Strengths of National Respiratory Pandemic Plans

**Figure 2.**
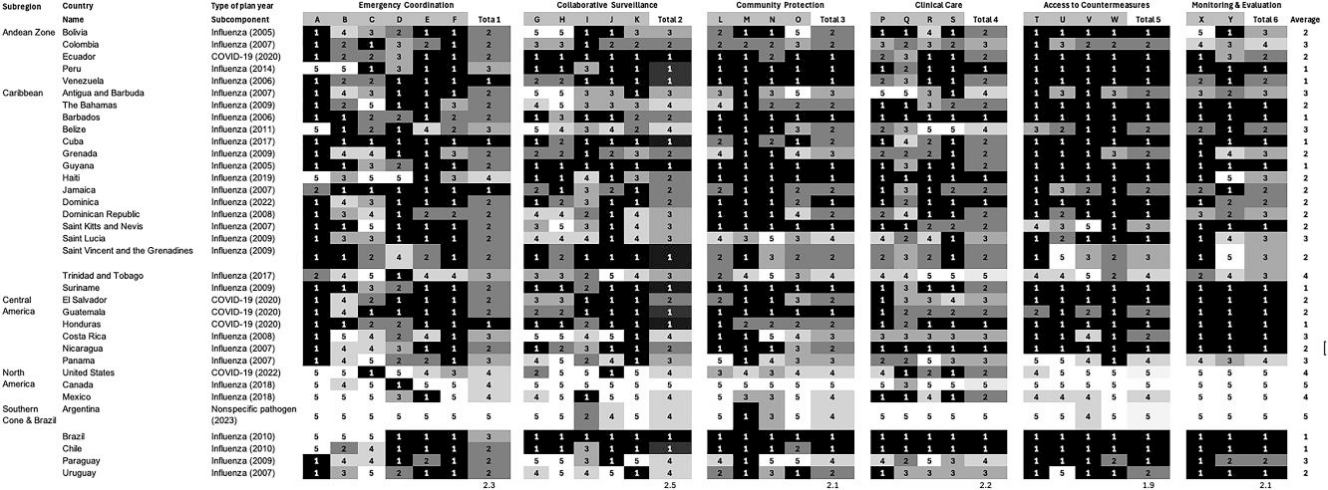
A heatmap analysis by pandemic checklist components and subcomponent integration by country and subregion. Emergency coordination: (A) planning; (B) policy, legal and normative instruments; (C) coordination; (D) financing; (E) human resources; (F) guiding principles, gender, and ethical considerations. Collaborative surveillance: (G) surveillance: overarching system considerations; (H) surveillance: early detection and assessment; (I) surveillance: monitoring circulating pathogen and use human health interventions; (J) laboratory; (K) One Health/zoonotic disease: collaborative efforts. Community protection: (L) public health and social measures: overarching; (M) public health and social measures: community; (N) border health and points of entry; (O) risk communication and community engagement. Clinical care: (P) health service provision: continuity of essential health services; (Q) health service provision: case management; (R) infection prevention and control; (S) safe management of a dead body. Access to countermeasures: (T) health emergency response: emergency logistics and supply chain management; (U) equitable access, needs-based allocation, and medical countermeasures deployment for pandemic products such as vaccines and antivirals—NDVP planning; (V) health emergency response: essential medicines, products, and materials; (W) health emergency response: research, development, and innovation; (X) monitoring and evaluating, testing, and revising plans: monitoring and evaluation; (Y) testing and revising plans. PP score: 1 = not mentioned, not described (0%); 2 = briefly mentioned in some part of the plan (25%); 3 = described with limitations (some of the criteria of the subcomponent are missing) (50%); 4 = fully described for national level (75%); 5 = described for all levels (national, provincial, and local) and/or updated based on the latest recommendations (100%). The average country scores were rounded to the nearest whole number, for ease of reading. Abbreviations: COVID-19, coronavirus disease 2019; NDVP, national deployment and vaccination plan; PP score, Pandemic Plans score.

**Figure 3.**
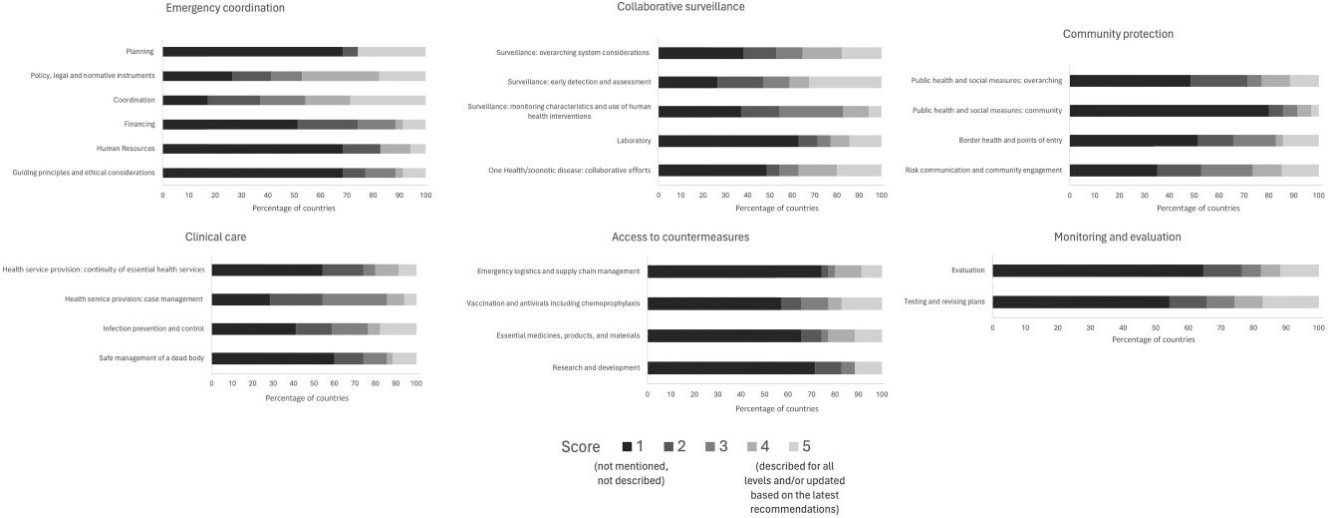
Relative proportion of plans within each scoring category by component and subcomponent of the pandemic checklist (regional summary). Pandemic Plans score (PP score): 1 = not mentioned, not described; 2 = briefly mentioned in some part of the plan; 3 = described with limitations (some of the criteria of the subcomponent are missing); 4 = fully described for national level; 5 = described for all levels (national, provincial, and local) and/or updated based on the latest recommendations.

The emergency coordination component of the plan had an average score of 1 or 2 (not mentioned or only briefly mentioned) in 24 (69%) of the 35 country plans (Figure 2). Only 5 (14%) of the plans fully described this component, of which 1 (3%) provided a comprehensive description across the levels (national, provincial, and local; score of 5). Notably, 24 (69%) plans did not mention lessons learned from previous pandemics, a crucial aspect of the planning subcomponent (Figure 2 and Figure 3). Within the community protection component, specifically the public health and social measures subcomponent was another weak area, with 28 (80%) plans scoring only a 1 (Figure 2 and Figure 3).

Significant gaps were also identified in the access to countermeasures component, which had the lowest average PP score of 1.9, with 27 (77%) plans scoring a 1 or 2 (Figure 2). In particular, the largest deficits were observed in the subcomponents related to health emergency response: emergency logistics and supply chain management (26 plans scored 1, 74%) and health emergency response: research, development, and innovation (25 plans scored 1, 71%) (Figure 2 and Figure 3).

Importantly, for the component monitoring and evaluation the average PP score was 2.1 with 21 (60%) plans scoring 1 or 2, meaning that they did not mention (43%) or only briefly mentioned (17%) testing, monitoring, and evaluation actions. This indicates a critical gap in testing, evaluating, and revising plans.

Despite these weaknesses, there were notable strengths in several areas. Collaborative surveillance was the component that was most frequently described in detail with an overall average PP score of 2.5 across all countries; 16 (46%) plans had an average PP score ≥3, and 9 (26%) plans scored an average of ≥4 (Figure 3). In particular, the subcomponent on surveillance: early detection and assessment was described in 19 (54%) plans (scores ≥3) and described in detail in 15 (43%) of plans (scores ≥4) (Figure 2).

While there were gaps in the emergency coordination component overall, there were some subcomponents within this area that demonstrated relative strength. A high proportion of plans scored a 3, 4, or 5 in the areas of coordination (22 plans, 63%) and policy, legal, and normative instruments (21 plans, 60%) (Figure 2).

Countries such as Canada and Argentina had the highest average scores, while countries like Barbados, Guyana, Suriname, and Honduras presented lower average scores. However, the overall scores of the plans in the countries across the region varied, highlighting areas for improvement and inconsistent performance (Figure 2).

### Concordance Between Respiratory Pandemic Plans and 2023 SPAR Indicators

**Figure 4.**
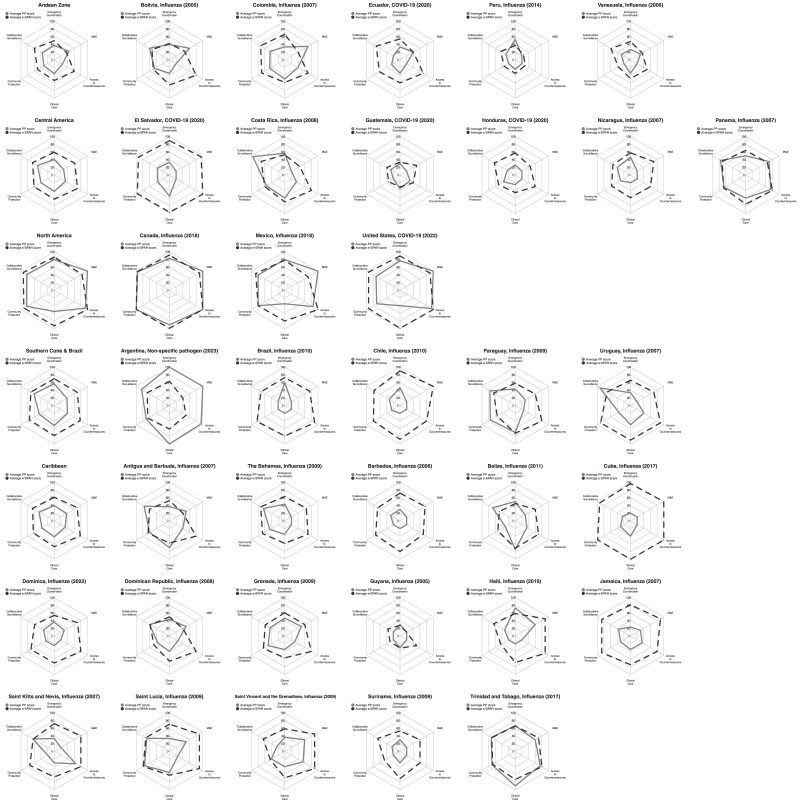
Alignment of PP and average 2023 SPAR for each pandemic checklist component by country. The e-SPAR scores shown represent the average of the indicator scores for each capacity reported in SPAR 2023. The PP scores shown represent the average of the subcomponent scores for each component of the pandemic checklist based on PRET Module 1. For the PP scores, a score of 1 (0%) was given if the subcomponent was not mentioned, not described; a score of 2 (25%) if it was briefly mentioned in some part of the plan; a score of 3 (50%) if it was described with limitations (some of the criteria of the subcomponent are missing); a score of 4 (75%) if it was fully described for national level; and a score of 5 (100%) if it was described for all levels (national, provincial, and local) and/or updated based on the latest recommendations. Abbreviations: e-SPAR, electronic State Parties Self-Assessment Annual Reporting Tool; PP score, Pandemic Plans score; PRET, Preparedness and Resilience for Emerging Threats.

Figure 4 shows radar graphs of PP scores and SPAR scores for each component in each country, with countries grouped by subregion. Generally, the SPAR scores tended to be higher than PP scores across most components. The magnitude of the difference between the PP and the SPAR scores varied for the different components for a given country, and occasionally, as in the case of Argentina, the pattern was reversed with the PP score exceeding the SPAR score. Canada, Guatemala, Panama, Peru, and Trinidad and Tobago had closely aligned PP and SPAR scores, with scores being ≥50% for all components; in the case of Guatemala, most of the scores were aligned except for 1 (monitoring and evaluation).

There were also some cases where 1 component's score was much higher than the other components. For example, emergency coordination had an elevated PP score compared to the scores of the other components in Brazil, and so did collaborative surveillance in Uruguay and Costa Rica.

## DISCUSSION

Our findings show that national pandemic preparedness and response plans in the Americas region are not adequately aligned with current global guidance (PRET Module 1 and the pandemic checklist). This partial alignment is understandable, given that most plans were evaluated based on guidance that did not exist at the time of their development. However, this gap presents a crucial opportunity to incorporate lessons learned from previous pandemics and adapt these plans to better respond to emerging threats such as influenza and COVID-19 [16]. Although this study did not include a stratified analysis by the year of plan publication, future studies may benefit from adopting this approach to gain a deeper understanding of how lessons from past pandemics influence pandemic preparedness, as well as the quality and components of the plans.

On a more positive note, this partial alignment also suggests that the available plans can serve as a foundation for strengthening preparedness capacities; in other words, countries do not need to begin from zero to update their plans. Countries can refer to the PRET initiative, which is a helpful resource that provides targeted guidance, training, simulation exercises, and other resources to support countries in updating national pandemic plans. PRET Module 1 also includes steps for organizing the planning process, a suggested outline for the plan, and describes elements to improve existing plans [8]. Findings from this study could help countries identify the areas to strengthen when updating their plans.

One area that would need revising in most plans is emergency coordination, which on average had the weakest scores. This is particularly true for the subcomponents of planning, financing, human resources and guiding principles and ethical considerations. Many plans revised in our study had yet to fully integrate emergency coordination in a robust, scalable, multisectoral and multilevel manner. This neglect of the emergency coordination component may affect planning, budget allocation, and coordination at all levels. Strengthening this component will also ensure legal backing for plans and for keeping plans current via systematic updating mechanisms built into the emergency coordination component of the plans, as espoused by PRET Module 1.

Our findings also indicated that most plans were written in response to an emergency, indicating a reactionary approach to pandemics rather than a preparatory approach, which is well recognized historically [17]. Indeed, when the timing of plan publication was examined more closely, we noted that most plans were published in response to either the 2009 influenza A (H1N1) pdm09 pandemic or the COVID-19 pandemic. Moreover, 25% of the plans initially identified were excluded for only including response components rather than provisions applicable to long-term preparedness.

Definitive recommendations from recognized authorities on updating current plans could help to address this issue. PRET emphasizes preparation as well as response, with preparedness being key in the interpandemic period. Preparedness is especially important given the increasing frequency of outbreaks of respiratory pathogens with pandemic potential and the increasing frequency and severity of pandemics caused by respiratory pathogens [18, 19]. In addition to addressing preparedness, given that contexts change over time, it is crucial that plans remain current. The absence of subcomponents directly related to maintaining preparedness as a dynamic state rather than as a static position recorded at the time of plan publication is especially apparent in our findings, with the overall low PP scores for the monitoring and evaluation component.

One of the areas with strong description in the plans examined was the collaborative surveillance component. This strength could be linked to the efforts of the global collaboration initiatives such as the Global Influenza Surveillance and Response System (GISRS) in terms of surveillance, laboratory coordination, influenza collaborating centers, as well as the collaborative work of the SARInet network, established in the Americas since 2014. This is a theoretical connection and requires further study.

The assessment of concordance between SPAR scores and PP scores revealed a tendency among countries to have higher SPAR scores than PP scores. The higher SPAR scores than PP scores may also be partly explained by the self-reported nature of SPAR scores, which makes them subject to perception bias. Additionally, the SPAR scores are reported annually, giving them the ability to change over time, in contrast to the static nature of the pandemic preparedness plan, which reflects a single point in time. Changes in SPAR scores from 2019 to 2020 were examined by Satria et al in 2022, and factors such as increased awareness and rigor with self-evaluation and limited resources were among the factors described to explain these changes [20].

Further studies that use qualitative research to analyze the processes and thinking behind both pandemic preparedness and response plans and the production of the SPAR scores may be useful. The disparities between these scores may also be affected by the challenge of countries not updating their plans regularly, thereby having outdated plans that do not reflect improvements in their capacities to prepare for and respond to pandemics and health emergencies. There are currently no global recommendations for the frequency of plan updates, but these could be developed upon further evidence and discussion.

WHO's approach to preparing for and responding to pandemics has evolved over the past from idealistic recommendations to practical guidance, reflecting a crucial recognition of the historical gap between recommendations and actual on-the-ground core capacities such as those required under the IHR (2005) [21]. This shift towards more pragmatic guidelines aims to better align planning with operational reality, emphasizing realistic and actionable steps that are more in line with what countries can implement given their current capabilities and resources.

Our study has several limitations. The first limitation is missing information in secondary data, as some plans lacked annexes and some links referenced within the documents were no longer functional, or the plan was unavailable (not shared or not available online). It is also possible that certain preparedness actions, including actions involved in emergency coordination, have been carried out but are not reflected in the documented preparedness plans. The second limitation is the variability in scope, detail, and quality between pandemic preparedness and response plans across countries, making comparisons between plans challenging. Additionally, this study is not exhaustive, and it does not imply that countries do not have more up-to-date plans or are not actively working on plan updates. Finally, the 25 tracking questions developed and used for this analysis is a novel tool that has not been tested. Further studies should focus on testing and refining this tool to ensure its accuracy and reliability in various contexts. The scoring of plans using the tool was also somewhat subjective, despite efforts to reduce bias by using a single dedicated primary reviewer how had not previusly exposed to these plans.

We recommend that all countries update their preparedness plans regularly, particularly the plans that do not reflect lessons learned from recent respiratory pathogen pandemics. For standardization, it may be useful for global guidance to be issued regarding the frequency of updating pandemic preparedness and response plans. It is also crucial that countries test their plans and include monitoring and evaluating activities within the plan, thus ensuring that capacity gaps and areas for improvement are identified.

We reiterate that most plans examined could form a foundation for improvements. We suggest, therefore, that countries explore developing a plan that addresses the most prominent gaps identified in this study between the plans and the PRET Module 1 and its associated pandemic checklist and SPAR. Specifically, countries may benefit from strengthening the emergency coordination component and the following subcomponents within the access to countermeasures component: health emergency response: emergency logistics and supply chain management; and health emergency response: research, development and innovation.

To enhance future preparedness, we recommend that national pandemic plans align with IHR and incorporate mechanisms for monitoring public health and social measures, updating national laws as needed, supporting vulnerable groups, and conducting stress tests for coordination [22]. Furthermore, centralizing relevant information and ensuring that all parties are informed of their roles and responsibilities will improve coordination during emergencies. This approach will help ensure that preparedness is maintained as a dynamic state, addressing gaps in current plans and enhancing overall response capabilities.

Certain preparedness actions, such as decision algorithms, may not be comprehensively documented in official plans. We recommend centralizing relevant information to improve stakeholder coordination and ensure clear role definition in future pandemics. This will ensure that all parties are clearly informed of their roles and responsibilities in future pandemics. Moreover, we encourage countries to ensure the integration of relevant specific plans, such as the National Deployment and Vaccination Plan, as annexes to their pandemic plans to ensure more effective coordination and a comprehensive response.

Finally, given that there is slightly improved coverage of emergency coordination in more recent plans, countries with plans updated within the past 5 to 7 years could consider reviewing and building on this component for their updated plans. It is, however, important that all countries assess their individual plans against current benchmarks and PRET guidance to address the specific gaps.

## CONCLUSION

Given the gaps identified between current plans and the global standards espoused by the PRET Module 1 initiative, which incorporates lessons learned from the COVID-19 pandemic, it is recommended that countries build on the strengths of their national pandemic preparedness and response plans and update them using PRET module 1. This will support countries to advance the capacities required by the IHR. Aligning plans with the latest international guidelines, implementing periodic monitoring and evaluation mechanisms, and strengthening multisectoral and multilevel coordination are key steps for a more effective response to future pandemics and for contributing to the resilience of public health systems in the region.

## Supplementary Data

Supplementary materials are available at *The Journal of Infectious Diseases* online (http://jid.oxfordjournals.org/). Supplementary materials consist of data provided by the author that are published to benefit the reader. The posted materials are not copyedited. The contents of all supplementary data are the sole responsibility of the authors. Questions or messages regarding errors should be addressed to the author.

## Supplementary Material

jiaf047_Supplementary_Data

## Notes

***Acknowledgments.*** We extend our deepest gratitude to the SARInet plus network for contributing to the regional and global respiratory virus surveillance through Global Influenza Surveillance and Response System/World Health Organization (WHO). We are grateful for the support of personnel at Pan American Health Organization (PAHO) headquarters and PAHO/WHO Subregional and Country Offices for the technical cooperation provided in the region to all respiratory viruses' pandemics. A special thank you to our PAHO colleagues that have been part of the Influenza Team for their collaboration throughout this journey, especially to Dr Jorge H. Jara, Dr Evelyn Balsells, and Luis Fernando Quesada. We would also like to express our gratitude for collaboration and support to the network from the WHO Collaborating Centers at US Centers for Disease Control and Prevention (CDC). Finally, we would like to thank all the donors to PAHO, including US CDC, US Department of Health and Human Services, Wellcome Trust, and WHO.

***Author contributions.*** A. P. V. R. and I. W. contributed study conception and design. A. P. V. R., I. W., T. M., and A. G. performed data collection. A. P. V. R., I. W., S. M., O. A., and M. E. B. R. performed analysis and interpretation of results. A. P. V. R., I. W., O. A., M. E. B. R., S. H., S. G., H. L., and B. M. prepared the draft manuscript. All authors critically reviewed the results, and the draft and final versions of the manuscript for intellectual content.

***Financial support.*** No financial support was received for this work.

***Supplement sponsorship.*** This article appears as part of the supplement “Advancing Respiratory Virus Surveillance and Preparedness in the Americas: A Decade of SARInet's Impact,” sponsored by the Pan American Health Organization.

***Potential conflicts of interest***. All authors: No reported conflicts of interest. All authors have submitted the ICMJE Form for Disclosure of Potential Conflicts of Interest. Conflicts that the editors consider relevant to the content of the manuscript have been disclosed.
