# Supplementary material for: Pandemic Preparedness: Analyzing National Plans for Respiratory Pathogen Pandemics in the Americas Region
Source: J Infect Dis. 2025 Mar 10;231(Suppl 2):S90–9. doi: 10.1093/infdis/jiaf047 (PMC11892002; doi:10.1093/infdis/jiaf047)
Supplement: jiaf047_Supplementary_Data [file jiaf047_supplementary_data.docx]

**Supplementary materials**

**Table S1. Formulation of the tracking questions by pandemic checklist’ subcomponent**

| **Tracking question for Emergency coordination** | **Pandemic checklist’ Subcomponent** | **Actionable area to improve the plan** |
| --- | --- | --- |
| Does the country plan mention lessons learned from past epidemics and pandemics of respiratory pathogens such as COVID-19, pandemic influenza A H1N1 in 2009? | Planning | Describe lessons learned from past epidemics or pandemics using as a guide the topics mentioned in Annex 5 of the PRET Module 1 |
| Does the country plan explicitly mention legal and normative instruments or policies that support pandemic preparedness and that were revised based on lessons learned from past epidemics or pandemics of respiratory pathogens such as COVID-19, pandemic influenza A H1N1 in 2009? | Policy, legal and normative instruments | Include a chapter dedicated to describing legal and policy considerations as in the suggested pandemic preparedness plan template for Section B (PRET Module 1, pg. 63) |
| Does the country plan define the multisectoral coordination mechanism (pre-existing or established in the plan)? | Coordination | Include a description of emergency coordination systems and procedures. (use as an example the proposed content in Part C (a), PRET Module 1, p 65). |
| Does the country plan contain or describe a budget or funding mechanism for pandemic preparedness and response? | Financing | Include a chapter on "funding for multisector preparedness and response." (PRET Module 1, p 64) |
| Does the plan contain or refer to a staffing plan for response to public health emergencies? | Human Resources | Include reference to an annexed or separate document that contains details of a strategic plan for workforce surge at all levels of the health systems, including clinical, laboratory, and public health workforce. (PRET Module 1, pg. 33-45; pg. 64). |

| **Tracking question for collaborative surveillance** | **Pandemic checklist’ Subcomponent** | **Actionable area to improve the plan** |
| --- | --- | --- |
| Does the plan explicitly reference ethical considerations related to vulnerable populations? Considerations include equitable access to life saving measures, for example. | Guiding principles and ethical considerations | Include explicit descriptions of ethical considerations that went into the planning process and how these will be factored into plan activation. (PRET Module 1, pg. 63). |
| Does the plan include a detailed description of the structure and functioning of the communicable disease surveillance system? | Surveillance: overarching system considerations | Include a chapter on Collaborative Surveillance with detailed description of surveillance mechanisms including One Health coordination, signal detection and verification, data and information management, and integration of data from multiple sectors. (PRET Module 1, pg. 65) |
| Does the plan describe and early warning surveillance system or mechanism for detecting respiratory virus events? | Surveillance: early detection and assessment | Include a sub-section of the plan that defines triggers for plan activation and the decision-making roles. (PRET Module 1, pg. 69) |
| Does the plan describe or make reference to guidance that describes the risk assessment processes and roles? | Surveillance: Monitoring characteristics and use of human health interventions | Include a subsection that describes triggers and procedures for shifting between operational stages, including risk and situational assessment protocols. (PRET Module 1, pg. 69) |
| Does the plan describe protocols for the referral and transportation of specimens in the event of a pandemic? | Laboratory | Include a section that describes how specimens will be transported or shipped to referral laboratories nationally or internationally, as well as how quality and biosafety will be ensured (PRET Module 1, pg. 65) |
| Does the plan describe procedures to coordinate surveillance across the human, animal and environmental health sectors for respiratory pathogens? | One Health/Zoonotic Disease: Collaborative Efforts | Include a description of the national and subnational mechanisms in place or to be implemented across the human, animal and environmental sectors (One Health) for pandemic preparedness and response (PRET Module 1, pg. 65) |

| **Tracking question for community protection** | **Pandemic checklist’ Subcomponent** | **Actionable area to improve the plan** |
| --- | --- | --- |
| Does the plan include criteria and procedures for implementing PHSM, including guidelines for when and how these measures should be put in place and lifted? | Public Health and Social Measures: over-arching | Include procedures for delivering PHSM and describe how PHSM will be considered (PRET Module 1, pg. 66) |
| Does the plan include guidance for schools and businesses to create plans for implementing PHSM? | Public Health and Social Measures: Community | Include measures for the continuation of education and learning, and to sustain other essential services (PRET Module 1, pg. 66) |
| Does the plan describe the triggers for implementing travel-related public health measures at points of entry (e.g. risk communication to travelers, IPC practice, surveillance and case management, transporting essential supplies)? | Border health and points of entry | Include procedures for maintaining travel and trade, such as how risk communication and risk mitigation measures will be designed and delivered to travelers (PRET Module 1, pg. 66) |
| Does the plan establish or account for a public health communication unit that can perform outreach across a variety of relevant platforms and to a diverse set of audiences? | Risk communication and community engagement | Include information on what two-way communications mechanisms will be used and how misinformation and disinformation will be monitored and addressed (PRET Module 1, pg. 66) |

| **Tracking question for clinical care** | **Pandemic checklist’ Subcomponent** | **Actionable area to improve the plan** |
| --- | --- | --- |
| Does the plan contain procedures for maintaining essential health services during a respiratory pathogen pandemic, such as facility-level operational plans for emergencies? | Health service provision: continuity of essential health services | Include procedures for how essential individual and population-based services will be maintained (PRET Module 1, pg. 67) |
| Does the plan include mechanisms to regularly review and update case management protocols? | Health service provision: case management | Include a section that describes how clinical management guidelines will be updated and rapidly disseminated |
| Does the plan include procedures for IPC during a respiratory pathogen pandemic, such as provision of PPE and other IPC supplies, WASH in health facilities, and protections for health workers? | Infection prevention and control | Include a section describing how health and care workers, patients and communities will be protected, particularly regarding: • access to infection, prevention and control methods, materials and training including water, sanitation and hygiene capacities, as well as occupational health • adequate WASH services in health facilities (PRET Module 1, pg. 67) |
| Does the plan account for excess mortality, including procedures for safe management and dignified disposal of dead bodies? | Safe management of a dead body | Include a description of how facilities will be scaled up for management of burials with safe and dignified practices (PRET Module 1, pg. 67) |

| **Tracking Question for access to Countermeasures** | **Pandemic checklist’ Subcomponent** | **Actionable area to improve the plan** |
| --- | --- | --- |
| Does the plan describe a supply chain management system that can scale up operations during a respiratory pathogen pandemic? | Health emergency response: emergency logistics and supply chain management | Include the following information:  - Descriptions of national stockpiles or access to an international stockpile that are implemented to support emergency response needs - Linkages to upstream (global and regional) supply chains and delivery  - Systems for downstream (in-country) supply chains and delivery be ensured (PRET Module 1, pg. 68) |
| Does the plan include actions for procuring and deploying vaccines during a respiratory pathogen pandemic? | Equitable access, needs-based allocation and medical countermeasures deployment for pandemic products such as vaccines and antivirals – NDVP planning | Include the following information: - Describe the systems and procedures for a national vaccination and deployment plan - How access to countermeasures will be maximized through global, regional or country level pre-negotiated agreements (PRET Module 1, pg. 68) |
| Does the plan include actions for procuring and deploying essential medicines, products, and materials during a respiratory pathogen pandemic? | Health emergency response: essential medicines, products and materials | Include a pre-defined list of essential supplies including diagnostics and therapeutics for managing different categories of respiratory pathogens. Describe how access to countermeasures will be maximized through global, regional or country level pre-negotiated agreements (PRET Module 1, pg. 68) |
| Does the plan contain or refer to measures that support ongoing research and development for medical countermeasures relevant to a respiratory pathogen pandemic? | Health emergency response: research, development and innovation | Include a description of how an enabling environment for research and development will be ensured (PRET Module 1, pg. 68) |

| **Tracking Question for Monitoring and evaluation** | **Pandemic checklist’ Subcomponent** | **Actionable area to improve the plan** |
| --- | --- | --- |
| Does the plan define indicators for pandemic preparedness and establish mechanisms for monitoring preparedness before, during, and after pandemics? | Monitoring and evaluation | Include indicators and milestones for preparedness including for the planning process (PRET Module 1, pg. 63) |
| Does the plan include procedures for regular updates, such as implementing the recommendations generated from monitoring and evaluation exercises? | Testing and revising plans | Describe an approach for planning that includes regular updating of plan based on preparedness and response needs (PRET Module 1, pg. 63) |

**Table S2: List of the plans selected for each country with the year of publication/update**

| **Country** | **Type of plan** | **Year of publication/ update** | **Title of the plan identified** | **Source/Link** |
| --- | --- | --- | --- | --- |
| Argentina | Other | 2023 | Plan de preparación y respuesta para eventos de salud pública de importancia nacional o internacional con potencial epidémico y/o pandémico (ESPINI) | https://servicios.infoleg.gob.ar/infolegInternet/anexos/390000-394999/393594/res3846.pdf |
| Antigua and Barbuda | Influenza | 2007 | National influenza preparedness plan (draft) | PAHO's landscape surveys |
| The Bahamas | Influenza | 2009 | Influenza and other vaccine preventable diseases | PAHO's landscape surveys |
| Belize | Influenza | 2011 | Health facility response plan for influenza pandemic | PAHO's landscape surveys |
| Bolivia | Influenza | 2005 | Plan nacional para pandemia de influenza y gripe aviar | PAHO's landscape surveys |
| Brazil | Influenza | 2010 | Plano brasileiro de preparação para enfrentamento de una pandemia de influenza | https://www.gov.br/saude/pt-br/acesso-a-informacao/gestao-do-sus/articulacao-interfederativa/cit/pautas-de-reunioes-e-resumos/2005/novembro/1-flu_seminariorj_jarbasbarbosa.pdf |
| Barbados | Influenza | 2006 | Barbados national influenza pandemic preparedness plan | PAHO's landscape surveys |
| Canada | Influenza | 2018 | Canadian pandemic influenza preparedness | https://www.canada.ca/en/public-health/services/flu-influenza/canadian-pandemic-influenza-preparedness-planning-guidance-health-sector.html |
| Colombia | Influenza | 2007 | Plan de prevención y mitigación del impacto de la pandemia de influenza | https://www.orasconhu.org/documentos/Influenza_aviar_colombia.pdf |
| Chile | Influenza | 2010 | Plan nacional de preparación y respuesta para una pandemia de influenza | PAHO's landscape surveys |
| Costa Rica | Influenza | 2008 | Plan de preparación y respuesta en situación de pandemia de influenza | https://www.binasss.sa.cr/opac-ms/media/digitales/Plan%20de%20preparaci%C3%B3n%20y%20respuesta%20en%20situaci%C3%B3n%20de%20pandemia%20de%20influenza.pdf |
| Cuba | Influenza | 2017 | Plan para el enfrentamiento de la pandemia influenza a (h1n1) | PAHO's landscape surveys |
| Dominica | Influenza | 2022 | Dominica national plan for pandemic influenza and other respiratory virus with pandemic | PAHO's landscape surveys |
| Dominican Republic | Influenza | 2008 | Plan nacional de preparación y respuesta frente a la pandemia de influenza 2007-2008 | PAHO's landscape surveys |
| Ecuador | COVID-19 | 2020 | Plan de preparación y respuesta del Ecuador ante la COVID-19 | https://www.salud.gob.ec/coronavirus-covid19-ecuador/ |
| Grenada | Influenza | 2009 | National Influenza Pandemic Preparedness Plan | PAHO's landscape surveys |
| Guatemala | COVID-19 | 2020 | Plan para la prevención,contención y respuesta a casos de Coronavirus (COVID-19) en Guatemala | PAHO's landscape surveys |
| Guyana | Influenza | 2005 | National Influenza preparedness plan | PAHO's landscape surveys |
| Honduras | COVID-19 | 2020 | Plan para la contención y respuesta a casos de coronavirus (COVID-19) en Honduras | https://oiss.org/wp-content/uploads/2021/11/Honduras_Plan_Coronavirus.pdf |
| Haiti | Influenza | 2019 | Plan Haïtien de Préparation a une Pandémie d’Influenza Version révisée 2019 | PAHO's landscape surveys |
| Jamaica | Influenza | 2007 | National plan of action for influenza a (H1N1) vaccination | PAHO's landscape surveys |
| Saint Kitts and Nevis | Influenza | 2007 | Avian/pandemic influenza national preparedness plan | PAHO's landscape surveys |
| Saint Lucia | Influenza | 2009 | National Influenza Plan | https://nemo.gov.lc/Disaster-Management/National-Emergency-Management-Plan/National-Plans |
| Mexico | Influenza | 2018 | Plan nacional para la preparación y respuesta ante la intensificación de la influenza estacional o ante una pandemia de influenza | https://www.gob.mx/salud/cenaprece/documentos/plan-nacional-para-la-preparacion-y-respuesta-ante-la-intensificacion-de-la-influenza-estacional-o-ante-una-pandemia-de-influenza |
| Nicaragua | Influenza | 2007 | Plan nacional de respuesta frente al riesgo de influenza pandemica e influenza aviar | PAHO's landscape surveys |
| Panama | Influenza | 2007 | Influenza aviar y la pandemia de influenza | PAHO's landscape surveys |
| Peru | Influenza | 2014 | Plan nacional de preparación respuesta frente a una potencial pandemia de influenza | PAHO's landscape surveys |
| Paraguay | Influenza | 2009 | Plan nacional de preparación y respuesta a la pandemia de influenza | https://dgvs.mspbs.gov.py/files/documentos/01_07_2016_19_35_50_Influenza-Plan-Nacional-de-Respuesta-a-la-Pandemia.pdf |
| El Salvador | COVID-19 | 2020 | Plan nacional de preparación y respuesta ante el novel coronavirus (2019 nCoV), El Salvador 2020 | PAHO's landscape surveys |
| Suriname | Influenza | 2009 | National Pandemic Preparedness Plan | PAHO's landscape surveys |
| Trinidad and Tobago | Influenza | 2017 | Pandemic Influenza Preparedness and response plan | PAHO's landscape surveys |
| Uruguay | Influenza | 2007 | Plan nacional integrado de preparación para una pandemia de influenza Uruguay 2007 | https://www.gub.uy/sistema-nacional-emergencias/sites/sistema-nacional-emergencias/files/documentos/noticias/plan_nac_integr_preparac_pandemia_influenza_uruguay_2007_0.pdf |
| United States | COVID-19 | 2022 | National covid-19 preparedness plan | https://www.whitehouse.gov/wp-content/uploads/2022/03/NAT-COVID-19-PREPAREDNESS-PLAN.pdf |
| Venezuela | Influenza | 2006 | Plan nacional de vigilancia, prevención y control ante una posible pandemia de influenza aviar | PAHO's landscape surveys |
| Saint Vincent and the Grenadines | Influenza | 2009 | St. Vincent and the Grenadines Influenza Pandemic Plan | PAHO's landscape surveys |
